# A qualitative study examining the impact of COVID-19 on capacity for sustainability of tobacco control programs

**DOI:** 10.1186/s12913-024-10633-9

**Published:** 2024-02-16

**Authors:** Jessica Gannon, Sarah Moreland-Russell

**Affiliations:** Prevention Research Center, Brown School, Washington University in St. Louis, St. Louis, MO USA

**Keywords:** Sustainability capacity, Tobacco control programs, Program sustainability, COVID-19

## Abstract

**Background:**

The coronavirus (COVID-19) pandemic presented a significant stressor on the public health system in the United States. While we know the immediate effects of the pandemic on public health programming, no literature has examined the resultant long-term impact on programmatic capacity for sustainability. This paper aims to identify the impact that the COVID-19 pandemic had on state tobacco control program’s capacity for sustainability.

**Methods:**

From December 2018 to January 2022, we conducted 46 technical assistance calls with tobacco control program employees from 11 states. Calls were audio recorded and professionally transcribed. We analyzed calls (*n* = 20) that took place during the COVID-19 pandemic. Thematic analysis focused on the impact the COVID-19 pandemic had on tobacco control program’s capacity for sustainability.

**Results:**

We identified six domains of sustainability that were impacted by COVID-19: (1) *funding stability*; (2) *organizational capacity*; (3) *partnerships*; (4) c*ommunication*; (5) *strategic planning*; and (6) *program adaptation*.

**Conclusions:**

Our study is the first to identify the impact of the pandemic on capacity for sustainability of tobacco control programs. Having an understanding of COVID-19’s influence on these sustainability domains could help with future public health programming during significant public health events and emergency preparedness.

**ClinicalTrials.gov Identifier:**

NCT03598114.

**Registration date:**

Retrospectively registered 02-07-2018.

## Introduction

The coronavirus (COVID-19) pandemic presented a significant stressor on the public health system in the United States. In the emergent response to the pandemic, state and territorial health departments implemented aggressive measures to support coordinated pandemic mitigation efforts [1, 2]. Nested within state and territorial health departments, state-level public health programs reallocated resources, including staff and funding, to support the aforementioned mitigation efforts. In addition to having to comply with new strict public health measures, the reallocation of resources limited and, in some cases, prevented the implementation of fundamental public health programming [3–5] potentially threatening the continuation and sustainability of some programs.

We define capacity for sustainability as the presence of structures and processes which allow a program to leverage resources to effectively implement and maintain evidence-based policies and activities [6]. In addition, the capacity for sustainability can be broken down into eight main domains as defined by the Program Sustainability Framework [6]. These domains include: organizational capacity, program evaluation, program adaptation, communications, strategic planning, funding stability, environmental support, and partnerships. The literature has documented several factors that effect fundamental public health programming across several of these domains. For instance, Pascoe et al. documented the effect of workforce turnover (organizational capacity) on evidence-based program sustainability [7]. In addition, Trust For America’s Health (a policy and research organization), annually documents the effect of consistent underfunding (funding stability) of public health programs [8]. The Program Sustainability Framework has been applied to numerous public health programs due to its applicability across areas at the local, state, and national levels [6]. For example, Tabak and colleagues [9] used the Program Sustainability Framework to perform a qualitative study exploring differences in high- and low-capacity local health departments. Another study [10] used the domains of the Program Sustainability Framework to explore the factors influencing the sustainability capacity of a coordinated approach to chronic disease prevention in state and territory health departments using a mixed-methods approach. Their qualitative interviews indicated that leadership, communications, partnerships, funding stability, and policy change were perceived as keys to success of the transition to a coordinated approach to chronic disease management. In addition, state tobacco control programs have been encouraged to use the framework and its corresponding assessment tool to create their sustainability plans as required by the Centers for Disease Control & Prevention (CDC)’s Office on Smoking and Health [11].

Among tobacco control programs, the framework has been used to assist states in program sustainability planning [12]. This process has helped state level tobacco control programs determine which program components were necessary to maintain the program and sustain its benefits over time. Across these planning events, states have highlighted a number of domains in which they needed to focus their efforts, including communications, program adaptation and environmental support.

While we know the immediate effects of the pandemic on public health programming, no literature has examined the resultant long-term impact on programmatic capacity for sustainability. It is important to understand the threats to sustainability so that programs can better plan for and re-build their capacity for sustainability long term. This paper aims to identify the impact that the COVID-19 pandemic had on state tobacco control program’s capacity for sustainability. The findings from this study will help public health programs plan for and mitigate disruption across sustainability domains during future public health emergencies to ensure ongoing effectiveness and sustainability of their programs.

## Methods

The present work is part of a larger study titled the Plans, Actions, and Capacity to Sustain Tobacco Control (PACT) study. The PACT study sought to build capacity for sustainability of evidence-based state tobacco control programs (TCPs) through in-person action planning training and access to ongoing technical assistance from our research team [13]. Evidence-based state TCPs are defined as the comprehensive efforts implemented in each state to fulfill requirements outlined in the *Best Practices for Comprehensive Tobacco Control Programs—2014* [14]. This includes five main components: 1. State and community interventions; 2. Mass-reach health communication interventions; 3. Cessation interventions; 4. Surveillance and evaluation activities; and 5. Infrastructure, administration, and management. The in-person action planning trainings took place during 2018 and 2019. Each state participating in the training was asked to include TCP staff and stakeholders (i.e. advocates, coalition members, volunteers, community based organizations, academics, etc.) who can represent the many aspects of the comprehensive tobacco control program. During these trainings, participants actively engaged in developing a sustainability action plan specific to their state TCP. These sustainability action plans included domain-specific objectives from the Program Sustainability Framework and included time-sensitive activities to be carried out by present stakeholders. The plans were designed to be implemented during the two years following participation in the training. The trainings followed the same structure, but were tailored to each state based on the chosen domain for their action plan. Following the in-person training, state TCPs received on-going technical assistance to trouble-shoot the action plans they designed and provide resources to continue their progress. As part of the provided technical assistance, we conducted 46 calls with TCP employees from 11 states. These calls took place over the phone or through Zoom from December 2018 to January 2022. In addition, from April 2020 through January 2022, we conducted a qualitative phenomenology to assess the potential effects of the pandemic on sustainability planning. Specifically, we added, “*How has the COVID-19 pandemic impacted the overall sustainability of your TCP?*” and “*Have the challenges you’ve experienced in implementing your sustainability action plan been a result of the COVID-19 pandemic, or were they present prior to COVID-19?*”

We recorded audio of the calls and had them professionally transcribed through rev.com. We obtained informed consent from participants prior to recording the calls and received ethical approval for this study from the Washington University in St. Louis Institutional Review Board. All the methods included in this study are in accordance with the declaration of Helsinki.

### Study participants

The PACT study used four criteria to select state TCPs to participate in the randomized control trial: policy progress, resources, need, and previous participation in a sustainability action planning training. States were randomized into control or intervention by stratifying the 50 states in the US into quadrants based on the states’ needs (adult smoking rates) and their tobacco control policy environments (American Lung Association’s (ALA) smoke-free scores, 2015) [15]. Three states with different degrees of funding (percentage of CDC recommended funding level actually spent) [16] were selected from each quadrant. We then selected the closest match for each state based on the same three characteristics. Each pair was randomized into control (*n* = 12) or intervention (*n* = 12). One intervention state dropped out of the study, leaving eleven intervention states (AL, FL, HI, ID, IL, MI, MS, NH, NJ, OH, and UT). Vitale et al. [13] provides additional details about state selection and recruitment. For the purposes of this study, we focused on the ten intervention states who participated in technical calls during the COVID-19 pandemic. For our technical assistance calls with state TCPs, we asked to speak with the individual most knowledgeable about their state’s work on building capacity for sustainability. This was generally the TCP director or program manager.

### Technical assistance call interview guide development

The semi-structured technical assistance (TA) call interview guide focused on the progress, changes, and challenges experienced in implementing the sustainability action plan, as well as resources needed by states to move forward with their action plans. The TA call interview guide primarily included open-ended questions followed by specific questions to garner a more detailed response from participants. The TA call interview guide questions were refined with input from the research team. Prior to the calls, we provided participants with the TA call interview guide questions. A full list of TA call interview questions can be seen in Table 1.

**Table 1:** Technical assistance call interview questions

Topic	Question(s)
PSAT Comparison Snapshot	What thoughts or reflections do you have about your Program Sustainability Assessment Tool scores?
Sustainability Action Plan	Have you accomplished your action plan goal? Could you talk a bit about what progress you have made toward accomplishing your goal?
Sustainability Action Plan	Have you made any changes to your action plan since we last spoke (i.e., adjusted dates, responsibilities, etc.)?
Sustainability Action Plan	What has been your greatest challenge in trying to reach your goal? What challenges have you encountered while implementing the action plan?
Sustainability Action Plan	Have the challenges you’ve experienced in implementing your sustainability action plan been a result of the COVID-19 pandemic, or were they present prior to COVID-19?
Sustainability Action Plan	How has the COVID-19 pandemic impacted the overall sustainability of your TCP?
Sustainability Action Plan	As the result of the new cooperative agreement, do you plan on creating a new action plan or revising your current one?
PACT work group	Has your entire PACT work group met either virtually or in-person in the last year? If so, what was discussed?
PACT work group	Have there been any additional changes to your PACT work group since we last spoke?
Resources	Have you utilized any PACT resources since the last time we spoke?
Resources	Are there any resources you need from us that will be beneficial moving forward?

### Data analysis

We conducted a thematic analysis of the qualitative responses using a deductive approach, in which the authors referenced their codebook to guide the process. The codebook consisted of four parent codes and sixteen sub-codes. We used these codes to inform emerging themes. We uploaded the transcribed TA calls to NVivo 20 software for coding. Three researchers coded transcripts until they achieved acceptable levels of inter-rater reliability [17] (kappa = 0.72). The remaining transcripts were coded by a single coder who identified and summarized themes using an inductive approach. Themes were reviewed by another researcher to ensure that they worked in relation to the coded data. For the purposes of this study, we analyzed TA calls that took place during the COVID-19 pandemic (*n* = 20) and we focused on the primary codes regarding the impact the COVID-19 pandemic had on tobacco control program’s capacity for sustainability. We used the Program Sustainability Framework [6] to structure the final themes and sub-themes presented in this paper.

## Results

Each state received an average of 2 calls/year during their two years of participation in the study. The average number of transcripts for each state during the COVID-19 pandemic was 2 with a range of 1 to 3. Our calls lasted an average of 31 min. Main themes aligned with domains from the Sustainability Framework that emerged from TA calls regarding the impact COVID-19 had on tobacco control program’s capacity for sustainability: (1) *funding stability*; (2) *organizational capacity;* (3) *partnerships*; (4) *communication*; (5) *strategic planning*; and (6) *program adaptation*.

### Funding stability

Funding stability involves creating a stable financial base that withstands changes in economic and political cycles [6]. Our thematic analysis showed that state TCPs endured challenges with funding stability throughout the pandemic, including concern for future budget cuts and difficulty spending down their regular program budget due to COVID-19.

#### Concern for future budget cuts due to COVID-19

Respondents reported being concerned that their TCP budget would be reduced due to the pandemic. They shared feelings of apprehension around how their state would cover the increased costs associated with the pandemic.

…*COVID has changed so much and there are just so many unemployed and so much money is going toward that right now… But when the state is looking where to pay its bills, they look at special funds. Those are things that they might be able to cherry pick like, “Do we really need to do this?”*

#### Difficulty spending down regular program budget due to COVID-19

Respondents also reported having difficulty spending down their regular program budget because they were not carrying out their usual programming during the pandemic.

*The challenges of spending down your budget when you can’t engage people.*

Some states proactively created messaging to their state legislature about the importance of keeping their TCP funded despite having a surplus of money left over at the end of their fiscal year.

*I’ve been working with our bureau director and some of our other partners on messaging for this, just the importance of don’t cut funding. This was a special year, it was a different year, if there’s unspent funds or underspending when it comes to specific tobacco things, that’s not a reason to cut any funding from the programs.*

### Organizational capacity

Organizational capacity involves having the resources and support needed to manage your program effectively [6]. A number of our findings about the impact of the COVID-19 pandemic on TCPs aligned with the organizational capacity domain; specifically it being difficult to do tobacco control work due to staff working on COVID-19 duties; hiring freezes due to COVID-19; and COVID-19 impacting service delivery at the local level.

#### Difficult to do tobacco control work due to staff working on COVID-19 duties

Many state TCPs felt their organizational capacity was diminished as they focused their efforts on COVID-19 response work, rather than tobacco control work.

*And the tribes that we work with have been impacted pretty heavily. All the tobacco coordinators at one point in time were or still are in full COVID-19 response. And so their work has… There hasn’t been as much as there would normally be.*

*One of our epidemiologists has been assigned to COVID since March, full-time. And our second epidemiologists, we had two, was assigned full-time to COVID until just a couple of months ago. So a lot of our stats are down.*

#### Hiring freezes due to COVID-19

During the pandemic, some state health departments were on hiring freezes in an effort to curb discretionary spending within their states. Due to these hiring freezes, TCPs were not able to fill vacant positions and thus had lower organizational capacity to carry out their work.

*So, it’s kind of like a both ends situation. COVID presented us with a set of problems where we were on a hiring freeze, and I couldn’t hire for the position for quite some time. And I had to develop a work around to initiate a hiring process for this position, because it was very critical to our program.*

#### COVID-19 impacted service delivery at the local level

Other state TCPs reported the impact of the COVID-19 pandemic on organizational capacity was primarily at the local level, rather than the state level.

*I think COVID has impacted service delivery at the local level more than it has at the state level… Definitely we’re stretched a little bit thin… but on average, I would say it hasn’t really affected service delivery. At the local level where they are having to do a lot more of the contact tracing and COVID relief efforts, my understanding is that some health districts have been impacted more severely than others…*.

### Partnerships

The partnerships domain is defined as creating connections between a program and its stakeholders [6]. Partners play a vital role in program sustainability, by making connections for programs, serving as champions during adversity, and filling in gaps in services. Findings from our thematic analysis indicate challenges with the partnerships domain including local health department partners were pulled into COVID-19 response work; priorities of external partners were not on tobacco control; and difficulty keeping external partners engaged while being virtual.

#### Local health department partners pulled into COVID-19 response work

State TCPs noted that staff from their local health departments were assigned to work on COVID-19 response.

…*chronic disease was pulled in almost immediately into COVID response. And so, that impacted all of my staff and their ability to do this work, but a lot of our partners have been pulled in too. So, our local health department’s staff that typically we would be just paying to do tobacco work have been reassigned to COVID work… it definitely derailed the work over the last year honestly.*

#### Priorities of external partners were not on tobacco control

Additionally, respondents shared that it was difficult to engage their external partners because their priorities were not focused on tobacco control.

*It’s just more of a challenge now too with COVID because I know everybody has their own priorities, to keep those new partners engaged and active so we’re still working to do that. And somehow even in this COVID environment, revitalize the efforts of the [Organization] and get people energized and interested in participating again, so that’s an ongoing thing that we’re dealing with.*

#### Difficulty keeping external partners engaged while being virtual

Partner engagement also suffered due to the virtual work environment. Respondents reported it was difficult to maintain partnerships while working remotely.

*I think the hardest thing is maintaining partners in the virtual environment… people are feeling their partnership’s weakening… I think that’s really what there’s a real need for in terms of how to utilize the virtual environment and make it work for you to maintain your partnerships.*

### Communication

The communications domain was also impacted by the COVID-19 pandemic. In our review of themes associated with the communications domain, difficulty keeping tobacco control on the public health agenda as a priority and using communications as a strategy to maintain tobacco as a public health priority aligned with our findings.

#### Difficulty keeping tobacco control on the public health agenda as a priority

In addition to keeping tobacco control a priority among partners, respondents also reported having difficulty communicating that tobacco control was still a priority within public health despite the pandemic.

*…we are having a hard time, and I think that this is the sentiment across many states at the state and local level, with communication and communication messaging to keep tobacco control as a priority in the midst of COVID. With a lot of staff at the local level being reassigned to COVID related activities, and some of our partners shifting priorities to address COVID more than maybe some of their other chronic disease program, it’s becoming very difficult to stress the importance of tobacco control.*

#### Using communications as a strategy to maintain tobacco as a public health priority

To combat the lack of priority given to tobacco control, some state TCPs focused their communication efforts on the link between tobacco control and COVID-19.

*So, we’ve been doing quite a bit of media lately, but it’s been paid media. My team is very much so interested in elevating the message between the linkage with COVID and tobacco, because COVID has definitely been overshadowing, like I said, the chronic disease areas, and it’s almost as if tobacco isn’t even a risk factor. Right?*

### Strategic planning

The strategic planning domain is defined as using processes to guide a program’s directions, goals, and strategies [6]. One theme associated with this domain, delaying strategic planning due to COVID-19, aligned with our findings.

#### Delaying strategic planning due to COVID-19

Respondents reported that the COVID-19 pandemic pushed some TCPs to delay their strategic planning process. Respondents noted that it would be possible to carry out the strategic planning process virtually, but saw benefit in waiting until they could have their first meeting in-person.

*And I will also take a step back and say, we’re due to have our state tobacco plan updated and we’ve not pursued that because we really feel like it will be important to have a kickoff meeting for that that’s in person. We know you can do it virtually, but if we are willing to wait another six months or even year, we just feel like it’ll be so much more fruitful to be able to do that.*

### Program adaptation

In our review of themes associated with the program adaptation domain, difficulty adapting school programs; adapting program related communications; and planning for future program adaptation aligned with our findings.

#### Difficulty adapting school programs

Respondents reported challenges in adapting their school-based programs to meet the ever-changing academic schedules carried out during the pandemic.

*So it’s been difficult to maintain interaction with the youth in schools during this time, with the fluctuating schedules and the same thing for community engagement. I see that one with our community coalitions, they’re continuing to do their work and they’re following virtual formats wherever necessary, but that has been a challenge for us as well and we knew it would be a challenge.*

#### Adapting program related communications

Additionally, respondents reported needing to adapt communications within their program due to the COVID-19 pandemic. In particular, respondents shared needing to restructure how they carried out their meetings in a virtual work environment.

*I think the other piece that probably ties into what [Name] was just mentioning about, particularly since COVID, we’re having to rebuild how we have meetings and come together and communicate, and that’s something that we need to work on…*.

#### Planning for future program adaptation

Finally, respondents shared asking their partners to create contingency plans for their programs outlining adaptations they would make should the pandemic continue to disrupt their work.

*So one of the things that we did when we developed scopes for this past fiscal year, ‘21. We asked each of our funded partners to come up with a contingency plan because we knew that COVID was still going to be a challenge. And we were like, “Okay, if this is still the situation, how are you going to ensure that your deliverables are carried out next year?”*

## Discussion

This paper identifies the impact of COVID-19 on tobacco control program’s capacity for sustainability. Using qualitative interviewing and thematic analysis, we identified six sustainability domains outlined by the Program Sustainability Framework that were impacted by COVID-19: (1) *funding stability*; (2) *organizational capacity* (3) *partnerships*; (4) *communication*; (5) *strategic planning*; and (6) *program adaptation*.

Schell et al. [6] noted that the domains of the Program Sustainability Framework can be divided into internal domains (Organizational Capacity, Program Adaptation, Program Evaluation, Communications, and Strategic Planning) and external domains (Funding Stability, Political Support, and Partnerships). The internal domains are primarily managed by the program itself, while the external domains are impacted by factors outside of the program. Findings from our thematic analysis show that both internal and external domains were impacted by COVID-19. Our study also provides a real-world example of the interconnectedness of the sustainability domains, where an impact on one domain can lead to an impact on the other domains [10].

Within the organizational capacity and partnerships domains, our findings highlight the impact of the pandemic at the local level of TCPs. Many local health department partners were pulled into COVID-19 response work, which limited their ability to support and contribute to tobacco-related work. Respondents also acknowledged that challenges with organizational capacity were primarily being felt at the local level, rather than the state level. These findings align with the National Association of County and City Heath Officials’ (NACCHO) 2020 Forces of Change survey which found 82% of local health departments reassigned staff from various programs to support pandemic response activities [18]. The survey also found that 65% of local health departments saw a reduction in their provision of tobacco, alcohol, and other drug prevention services [18].

Findings related to the strategic planning domain are concerning, as strategic planning is critical to sustainability [6] and considered to be the “glue that holds sustainability efforts together” [19]. Without a strategic plan in place, programs risk losing focus on their direction and long-term goals. This can lead to a program that reacts only to the day-to-day demands of their work. Thus delayed strategic planning during COVID-19 could have long lasting impacts on program goals and future sustainability efforts.

The results related to the funding stability domain highlight the concerns related to the threat of funds being taken away and reallocated to other programs. While a threat to funding is not an uncommon event in tobacco control programming, this threat was distinct– funds were available, but were being resourced to address the pandemic. In addition, some tobacco control programs experienced difficulty in spending their programmatic budgets throughout the pandemic because regular programming was not occurring. These findings are unsurprising given the continued cuts in funding for overall prevention and public health programming [8]. The Federal government’s Prevention and Public Health Fund, which was originally created to expand and sustain the United States investment in prevention and public health programs [20], is at roughly half the funding level Congress should have provided due to the redirection of money to other programs and legislation [8].

While the majority of our findings point out the challenges to building capacity for sustainability among TCPs during the pandemic, our results related to the communications and program adaptation domains highlight the way TCPs pivoted to respond to these challenges. At the start of the pandemic, TCPs diverted resources to COVID-19 response. With priorities shifting to the pandemic, TCPs felt that they were being overshadowed by the broader public health crisis unfolding. In response, some TCPs developed public health messaging about the increased risk of COVID-19 as a smoker [21]. This strategy aligns with recommendations from Carter et al. [5] for TCPs to take the liberty to create contingency plans for their programs as the pandemic continued to disrupt programming. This forethought allowed them to plan for program adaptions should the pandemic continue to disrupt their work.

Two domains from the Program Sustainability Framework were not present in our data: environmental support and program evaluation. While our findings did not align with either of these sustainability domains, they still might have been impacted by the COVID-19 pandemic. For example, Kintziger et al. [22] studied the impact of COVID-19 response on the delivery of other public health services and found a significant decline (decrease of 36%) in the number of individuals in the public health workforce working in program evaluations during the pandemic as staff were shifted to COVID-19 response. We also know that public health programs were facing widespread pressure from outside forces, and a lack of public trust in public health occurred during the pandemic [23].

### Limitations

Our study has a few limitations. First, although the respondents in our study represented a diverse group of states with different levels of funding and policy progress, they make up only a fraction of TCPs in the United States. Therefore we may not have captured all impacts COVID-19 had on TCPs capacity for sustainability. We also had a limited number of TA calls with states during the pandemic. When the study first began, we carried out TA calls on a quarterly basis. During the early months of the pandemic (March 2020– November 2020), we paused TA calls in an effort to not over-burden state TCPs based on guidance from our practice advisory group. For that reason, we may not have captured the initial impacts of the pandemic on state TCPs capacity for sustainability. When we restarted our TA calls it was more difficult to engage some of the state TCPs and schedule calls, thus we had an unequal number of technical assistance calls with each state. There were also states who concluded their participation in the PACT study shortly after we restarted our TA calls. For these reasons, one state TCP’s experience during the pandemic may have been more evident than another state’s because we had more calls with them. Despite these limitations, we were able to capture the wide range of impact the pandemic had on TCP capacity for sustainability.

## Conclusion

Our study is the first to identify the impact of the COVID-19 pandemic on capacity for sustainability of tobacco control programs. Using the Program Sustainability Framework [6] we identified six domains of sustainability that were impacted by COVID-19: funding stability; organizational capacity; partnerships; communication; strategic planning; and program adaptation. Having an understanding of COVID-19’s influence on these domains could help with future public health programming during significant public health events and emergency preparedness. While this research was conducted with state TCPs, these results are likely applicable to other public health programs. Evidence indicates that the COVID-19 pandemic resulted in a significant disruption in public health and in health service delivery across the globe [24]. COVID-19 pressurized public health and other health systems stretching them beyond their capability to effectively implement public health services directed for the prevention and treatment of non-communicable diseases [25–27]. While state and local level programs are required to have an emergency preparedness response plan in place, the COVID-19 pandemic exposed some of the gaps in these plans. Using the sustainability framework as a guide, state and local planners can review the components of their plan and highlight areas specifically impacted by COVID-19 and at risk of fail during future response efforts. For instance, emergency preparedness and response plans should include a protocol for how to carry out other non-communicable preventative and curative services during an event. Based on our study results, the protocol should include components that address staffing, funding and stakeholders needed to effectively continue state public health programming. Future research should focus on examining how the pandemic impacted other chronic disease prevention program’s capacity for sustainability to understand whether similar results occurred across public health programs. Additionally, future research should identify how to mitigate disruption of these sustainability domains to ensure the ongoing effectiveness of public health programs.

## Data Availability

The datasets used and analyzed during the current study are available from the corresponding author on reasonable request.

## Declarations

### Human guideline or accordance statement

All the methods included in this study are in accordance with the declaration of Helsinki.
